# Multivalent Disabled Infectious Single Animal (DISA)-DIVA vaccine for all nine serotypes of African horse sickness virus (AHSV) is broadly protective in IFNAR (-/-) mice

**DOI:** 10.1186/s13567-026-01753-7

**Published:** 2026-04-16

**Authors:** Sergio Utrilla-Trigo, Luis Jiménez-Cabello, René G. P. van Gennip, Piet A. van Rijn, Javier Ortego, Eva Calvo-Pinilla

**Affiliations:** 1 Centro de Investigación en Sanidad Animal, CISA-CSIC, Valdeolmos, Madrid, Spain; 2 Wageningen Bioveterinary Research (WBVR), Lelystad, The Netherlands; 3 Department of Biochemistry, Centre for Human Metabolomics, North-West University, Potchefstroom, South Africa

**Keywords:** African horse sickness, safe vaccines, Disabled Infectious Single Animal, multiserotype efficacy, neutralizing antibodies

## Abstract

African horse sickness (AHS) is a highly severe, midge-borne disease of equids showing a mortality rate of > 90% in naive domestic horses. Outbreaks are caused by AHS virus (AHSV) and result in devastating economic losses in developing countries as equids play a crucial role in the agricultural industry and many households. The AHSV species encompasses nine serotypes showing no or limited cross protection, which is challenging regarding AHS vaccine development. Recently, and like bluetongue virus (BTV), the AHS Disabled Infectious Single Animal (DISA)-DIVA vaccine platform was developed and applied for all nine AHSV serotypes. The vaccine platform is based on a single deletion crucial for vaccine safety and affording differentiation of infected from vaccinated individuals (DIVA) testing. Before considering ethically debatable and expensive experiments in equids, DISA-DIVA vaccine candidates were evaluated here in the previously validated IFNAR (-/-) mouse model for AHS. A multivalent AHS DISA-DIVA vaccine containing DISA constructs for each of the nine serotypes was studied. This "DISA cocktail" was safe and protected mice against two virulent AHSV serotypes used in challenge infection studies. DISA multivalent vaccine induced serotype-specific neutralizing antibodies (nAbs) against all AHSV serotypes suggesting broad protection, as correlates of protection (CoP) based on nAb titers for studied serotypes can be extrapolated to others. These results now justify further experiments in horses to study safety, efficacy and DIVA by DISA cocktail vaccine. Importantly, the presented results for IFNAR (-/-) mice indicate that broad protection of equines by vaccination with DISA cocktail vaccine can be feasible.

## Introduction

African horse sickness (AHS) is a highly severe disease of equids showing >90% mortality in naïve horses and is designated as a category A notifiable disease by the World Organization for Animal Health (WOAH) [1]. The severity of AHS ranges from mild febrile illness to peracute forms that often results in sudden death without timely medical intervention [2, 3]. Most AHSV serotypes are endemic to sub-Saharan Africa and present a major threat to equids and the broader equine industry [4]. In developed countries, AHS outbreaks also lead to huge losses in the equestrian industry and have an enormous socio-emotional impact on owners of leisure horses. AHS has become a serious threat to countries with moderate climates where endemic *Culicoides* species are competent vectors of closely related orbiviruses.

The causative agent is African horse sickness virus (AHSV), a member of the *Orbivirus* genus, of which bluetongue virus (BTV) is the prototype orbivirus, within the *Sedoreoviridae* family [5]. Orbiviruses are non-enveloped, multiprotein-layered virus particles with a ten-segmented genome of double-stranded RNA (Seg-1 to Seg-10) [6, 7]. Each orbivirus species encompasses multiple serotypes. The segmented genome of orbivirus species enables exchange of genome segments that is well-known as reassortment or genetic shift [6, 8].

Like BTV and epizootic hemorrhagic disease virus (EHDV), AHSV is predominantly transmitted by *Culicoides* biting midges [9–12]. The habitat of competent midges is expanding due to various factors including climate change [13, 14]. Additionally, *Culicoides* species endemic in moderate climate are competent vectors of BTV [15–17] and more recently of EHDV [18, 19]. This implies that AHS-free countries also become at risk of AHS [20, 21]. Incursions into previously unaffected areas, such as parts of Europe, North Africa, and Asia [22–25], have reinforced the potential for global dissemination and the critical need for effective control strategies. Among intervention tools, vaccination is the most cost-effective method for managing vector-borne diseases such as AHS, especially in endemic regions experiencing re-introduction from wildlife reservoirs, and for disease-free countries poses a high risk of incursions.

The AHSV species encompasses nine serotypes showing no or low cross protection, which hampers vaccine development for AHS [26]. Serotype specificity is mainly determined by Seg-2 encoding outer shell protein VP2, the target of serotype specific neutralizing antibodies (nAbs) [27]. Currently used vaccines in Africa are primarily live attenuated vaccines (LAVs) [23]. LAVs have been derived by many cell passages of field strains of AHSV serotypes [28, 29] leading to point mutations that are likely scattered over different genome segments and differ between LAVs. Although LAVs still play a significant role in AHS control in endemic African regions, they have inherent drawbacks [30]. These include risks of incomplete attenuation (residual pathogenicity and viremia) and uncontrolled spread by midges and risk of genetic reassortment between LAVs or with field strains resulting in new pathogenic variants [31–33]. Due to these biosafety concerns, LAVs are limited or prohibited in non-endemic regions and have prompted alternative vaccine approaches. New vaccine candidates include VP2 subunit vaccines, recombinant viral vectors expressing AHSV proteins and virus-like particles [29, 34–36]. These approaches seek to improve vaccine safety and focus on strong immune responses, particularly against serotype specific immunodominant VP2 proteins.

Establishment of reverse genetics systems for BTV and AHSV, provides a powerful tool for development of virus-based vaccines for these WOAH-notifiable diseases, reviewed in [37, 38]. These approaches aim to combine the high efficacy of traditionally generated LAVs with significant advantages, like improved safety and the potential for differentiation of infected from vaccinated individuals (DIVA-principle) in serological tests, reviewed for BTV [39]. Likewise, virus-based vaccines for AHSV have been explored [38, 40, 41]. For example, previous studies demonstrated that an in-frame deletion in Seg-10 of a highly virulent AHSV-5 strain completely abolished viremia in ponies [42]. Moreover, this region was targeted to develop diagnostics to detect AHSV infected equines irrespective of their vaccination status [43].

Similar to the extensively studied BT Disabled Infectious Single Animal (DISA)-DIVA vaccine platform [39, 44, 45], the AHS DISA-DIVA vaccine platform has been applied for all nine serotypes [46]. Briefly, such DISA-DIVA vaccine platforms consist of eight genome segments of live attenuated vaccines. These backbones include a significant in-frame deletion of > 70 amino acid codons in Seg-10 expressing NS3/NS3a protein, which is not essential for in vitro virus replication. It is important to note that this deletion is solely responsible for the no virulence in mammalian hosts, for a double block on vaccine transmission by midges (DISA-principle), and is the target of DIVA diagnostics [41, 42, 47]. The genome constellation is completed by Seg-2 and Seg-6 expressing serotype-specific outer shell proteins VP2 and VP5, respectively. Advantageously, DISA-DIVA vaccines are produced in well-known cell lines as used in established vaccine production facilities. For AHSV, DISA-DIVA vaccine candidates for all nine serotypes have been designated as DISA1 to DISA9 based on their serotype [46].

Safety and efficacy of AHS vaccine candidates should be studied in the equine target host. However, such studies in horses require strong ethical evaluation and are costly. Similar to BTV and EHDV, mice deficient in the type I IFN (IFN-α/β) receptor (IFNAR (-/-) have been established as a reliable and validated alternative animal model for AHS [48, 49]. These transgenic IFNAR (-/-) mice are highly susceptible to AHSV infection and partially reproduces the disease pathogenesis observed in natural hosts [50–52]. Here we employed this experimental IFNAR (-/-) mouse model to evaluate the immunogenicity and protective capacity of a multivalent AHS DISA-DIVA vaccine. This preclinical trial will enable reduction of the number of horses required for vaccination trials to evaluate the profile of these DISA-DIVA vaccines in horses.

## Materials and methods

### Cell lines and viruses

Green monkey kidney cells (Vero) (ATCC, Cat. No. CCL-81) and baby hamster kidney cells (BHK-21; ATCC catalog No. CCL-10), were grown in DMEM supplemented with 2 mM glutamine, 100 U/mL penicillin, 100 µg/mL streptomycin, and 5% fetal calf serum.

AHS DISA-DIVA vaccines, named shortly DISA1 to DISA9 after their serotype, were generated by reverse genetics as described [46]. Briefly, the AHS DISA-DIVA vaccine platform is based on the LAV for serotype 4 containing the in-frame deletion of 77 amino acid codons in Seg-10 as previously generated by reverse genetics [40]. The genome constellation of the vaccine backbone is completed by Seg-2 and Seg-6 of the respective AHSV serotype to recover DISA1 to DISA9.

Viral stocks of DISA1 to DISA9, AHSV serotype 4 (Madrid/87) (AHSV-4) and AHSV serotype 5 (generated by reverse genetics) (rAHSV-5) [42] were produced by one passage in BHK-21 cells. Stocks were produced by infection of 80% confluent BHK-21 cells using a multiplicity of infection (MOI) of 0.1 and incubation at 37 °C for approximately 48–72 h. When a total cytopathic effect was observed, cells and supernatant were collected. Harvests were clarified by centrifugation (2000 rpm, 10 min) and supernatants were stored at −80 °C. Virus titers were determined by endpoint dilution and expressed as 10 log 50% tissue culture infective dose per milliliter (TCID_50_/mL). Multivalent AHS DISA-DIVA vaccine, here named "DISA cocktail vaccine", was formulated by combining equal amounts of DISA1 to DISA9. One dose of 200 µL DISA cocktail vaccine contained 9 × 10^5^ TCID_50_ DISA vaccine in total, corresponding to 10^5^ TCID_50_ per DISA vaccine.

Viral titrations were performed in Vero cell cultures by plaque assays and calculated in plaque-forming units (PFU) per milliliter. Ten-fold dilutions of viral stocks were incubated into Vero semi-confluent monolayers previously grown in 12-well plates. After 90 min incubation, an agar overlay (0.4% Noble agar in DMEM 10% FCS) was added and plates were incubated for 5 days at 37 °C in 5% CO_2_. Alternatively, to calculate TCID_50_ /mL, virus titration was performed in Vero M96 well plates using ten-fold dilutions, incubated for three days and fixed [53]. Plates were fixed with 10% formaldehyde and visualized with 2% crystal violet in MeOH.

### Animal experiments

Type I interferon receptor defective mice, IFNAR (-/-), on a 129 Sv/Ev background were bred in the animal care facility of the Department of Animal Reproduction at INIA-CSIC and housed under pathogen-free conditions at the biosafety level 3 (BSL3) animal facilities in the Animal Health Research Center (CISA-INIA,CSIC), Madrid (Spain), one week before the experiments.

**Figure 1:**
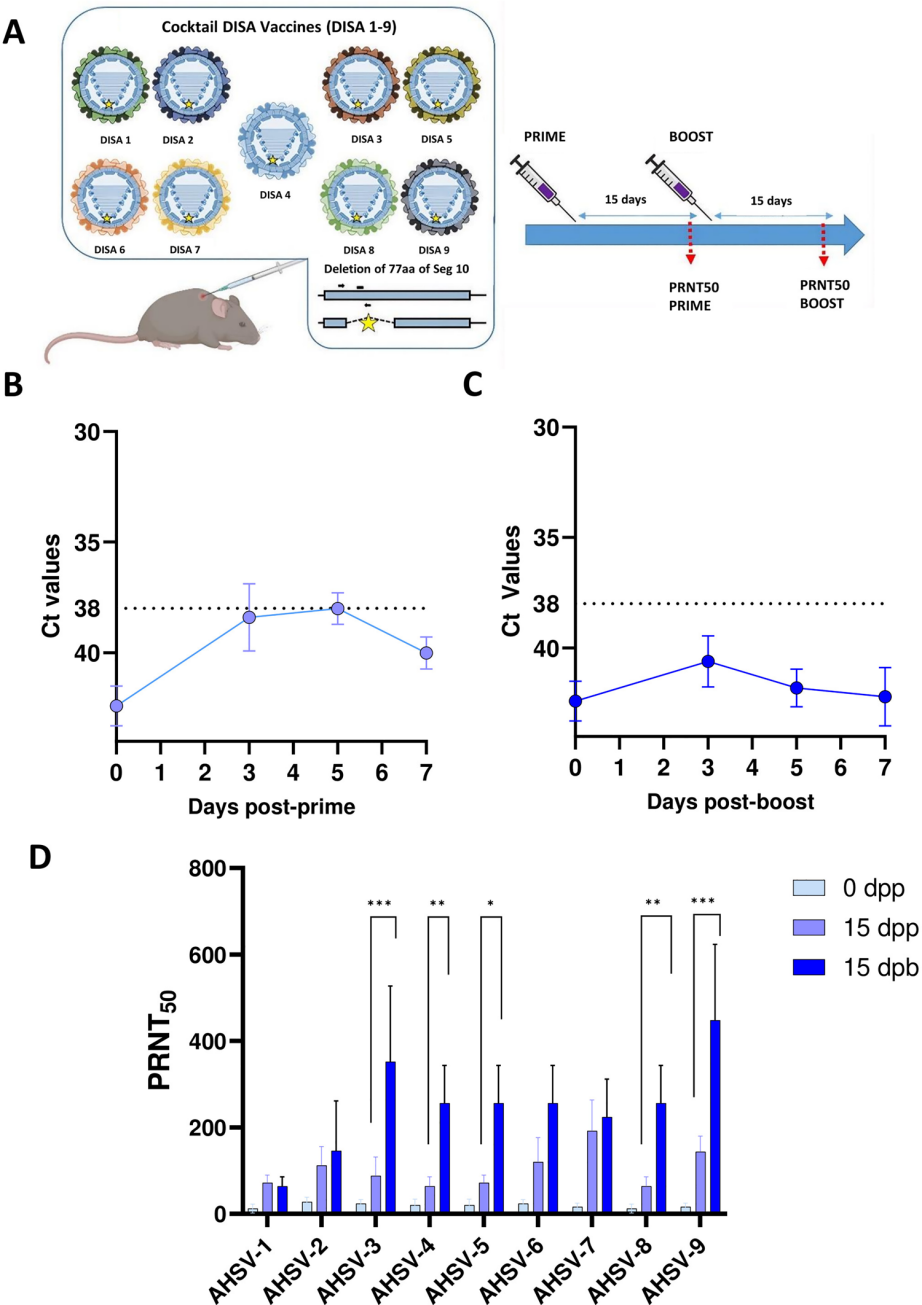
**Safety and immunogenicity of AHS DISA cocktail vaccine in IFNAR (-/-) mice.** **A** Experimental design to study safety and neutralizing antibodies after immunization with DISA cocktail vaccine. A group of IFNAR (− / −) mice (*n* = 5) was immunized with two doses of DISA cocktail vaccine containing equal amounts of DISA1 to DISA9. Sera were collected at days 15 and 30 of the experiment, after the prime and the booster immunization, respectively. **B** Blood samples were collected at 3, 5, 7, days post-prime or **C** post-boost for quantification of RNAemia by qRT-qPCR. **D** Plaque neutralization assays were performed to determine nAb titers against each of the nine AHSV serotypes at day 0 and 15 post-prime (dpp) and day 15 post-boost (dpb). Bars represent mean nAb titers for each serotype and the standard deviation is indicated by error bars. Two-way ANOVA with a post hoc Tukey test for multiple comparisons was used to calculated statistic differences (* = *P* < 0.05; ** = *P* < 0.01; *** = *P* < 0.001).

First, one group of mice (*n* = 5) was intraperitoneally inoculated with two doses of DISA cocktail vaccine 15 days apart to study safety and immunogenicity (Figure 1A). Animals were daily monitored and neutralizing antibodies were evaluated in sera at day 15 after prime and day 15 after boost. Viral RNA levels were tested in mice blood collected at 3, 5 and 7 days after each vaccination.

Second, three groups of mice (*n* = 5) were subcutaneously inoculated with different doses of rAHSV-5 (10^3^, 10^4^ and 10^5^ PFU/mouse) to determine the infective dose of challenge virus for future animal trials. Mice were daily monitored and 100 µL blood samples were collected at 3, 5, 7 and 10 days post-infection (dpi).

Third, four groups of mice (*n* = 5) were intraperitoneally vaccinated and subcutaneously challenged with virulent AHSV serotypes to study protection. Two groups were immunized with one dose and two groups with two doses of DISA cocktail vaccine administered two weeks apart. Then, two weeks after the last vaccination, two vaccinated groups, together with a control non-immunized group (*n* = 5), were infected with 10^6^ PFU of AHSV-4 or with 10^5^ PFU of rAHSV-5, respectively. After infections, animals were monitored for clinical signs twice a day. Daily clinical scores were calculated for each animal according to a list of clinical signs, such as weight loss, activity, hunching, respiratory alteration, severe conjunctivitis or any other condition that prevented food or water intake (Table 1). Individual mice were humanely euthanized when the humane ethical endpoint (HEP) was reached (>9). Blood samples were collected at indicated days post infection and were assayed for viral RNA and viremia.

**Table 1:** **Table of semi-quantitative clinical signs**

Clinical sign	0	1	2	3
Respiratory signs	Normal	Slight alteration	Moderate alteration	Marked alteration
Movement	Normal	Mild difficulty	Moderate difficulty	Severe difficulty
Hair appearance	Normal	Ruffled hair		
Conjunctivitis	Absence	Mild	Moderate	Severe
Weight loss	Mild (< 5%)	Moderate (5–10%)	Severe (11–20%)	Very severe (> 20%)
General appearance	Normal	Isolated animal (lethargy) without arched back	Inactive and isolated animal (lethargy) with slightly arched back	Inactive and isolated animal (lethargy) with severely arched back

Humane euthanasia of individual animals was carried out if the daily calculated clinical score was > 9 (humane ethical endpoint (HEP), or if weight loss exceeded 20%, or by severe difficulty in breathing and movement.

### Plaque reduction neutralization test (PRNT _50_)

Sera were tested for neutralizing antibodies using standard procedures for AHSV. Briefly, two-fold dilutions (from 1:4) of heat-inactivated mouse sera (56 °C for 30 min) were incubated with 100 PFU of AHSV for 1 h at 37 °C. Then, samples were inoculated into 12-well plates containing semi-confluent monolayers of Vero cells. Following incubation for 1 h, an agar overlay (DMEM-10%-FBS-0.4%-Noble Agar, Becton Dickinson, MD, USA) was added, and plates were incubated for 5 days at 37 °C in 5% CO_2_. Plaques were fixed with 10% formaldehyde and visualized with 2% crystal violet-MeOH. PRNT_50_ titers (nAb titers) were expressed as the reciprocal of the last serum dilution showing 50 percent reduction in plaque counts compared to wells without serum.

### Reverse transcription-quantitative real-time RT-PCR assay

Blood samples (50 μL) from mice were collected in EDTA tubes and used for RNA extraction with TRI Reagent (Sigma-Aldrich, St. Louis, MO, USA) following manufacturer protocol. AHSV RNA level (RNAemia) was quantitatively estimated by the real-time reverse transcription-quantitative PCR assay (RT-qPCR) assay [54]. Primers and probe were directed to a highly conserved sequence within segment 7 of the AHSV genome. Forward and reverse primer sequences are 5′-CCAGTAGGCCAGATCAACAG-3′ and 5′-CTAATGAAAGCGGTGACCGT-3′, respectively. The probe consisted of 5′-FAM- GCTAGCAGCCTACCACTA-MGB-3′ (Sigma-Aldrich, USA). Amplification conditions consisted of a first reverse-transcription step at 55 °C for 30 min, followed by 15 min at 95 °C, and 45 cycles of 15 s at 94 °C, and 1 min at 60 °C. Ct values of < 38 were considered positive.

### Viremia analysis by plaque assay

For the analysis of viremia by plaque assay, 50 µL of mouse blood were washed in PBS and centrifuged at 3000 rpm for 10 min. Thereafter, supernatant was removed, and pellet was lysed in 450 µL of sterile water for 2 min. Cell lysis was stopped by adding 50 µL of PBS-10X. Then, samples were inoculated into 12-well plates containing semi-confluent monolayers of Vero cells. Following 1 h incubation, an agar overlay (DMEM-10%-FBS-0.4%-Noble Agar, Becton Dickinson, MD, USA) was added and plates were incubated for 5 days at 37 °C in 5% CO_2_. Plaques were fixed with 10% formaldehyde and visualized with 2% crystal violet.

### Statistical analysis

Data was analyzed using the software GraphPad Prism version 8.0.1 (GraphPad, San Diego, CA, USA). Data from PRNT_50_ assays were analyzed using Mann–Whitney non-parametric test. Comparisons of mean responses between groups in the viremia and RNAemia analysis were conducted by multiple t test analysis using the Sidak–Bonferroni method. A *P*-value lower than 0.05 was considered statistically significant in all cases.

## Results

### Safety and immunogenicity of AHS DISA cocktail in IFNAR (-/-) mice

To evaluate safety and immunogenicity, a group of IFNAR (-/-) mice (*n* = 5) was immunized twice with the potential ‘DISA cocktail vaccine’ (multivalent AHS DISA-DIVA vaccine containing equal amounts of DISA1 to DISA9, 9 × 10^5^ TCID_50_/dose in total per mouse) (Figure 1A). Mice did not develop any clinical signs nor changes in behavior after immunizations at any time point of the experiment, which indicated that DISA cocktail vaccine is safe and well-tolerated in the mouse model. Moreover, safety of DISA1 to DISA9 and the DISA cocktail vaccine was confirmed by absence of viral RNA as measured by the highly sensitive RT-qPCR of blood samples collected at 3, 5 and 7 days after the first and second immunization. Mean Ct values of ≥ 38 were under the cut-off of the assay for all analyzed samples (Figures 1B, C), demonstrating the safety of the DISA cocktail vaccine as well as of individual DISA vaccines.

Blood collected on day 15 after the prime and boost immunizations with DISA cocktail vaccine showed serotype specific nAbs as determined by plaque assays with each of the nine AHSV serotypes (Figure 1D). Notably, nAb titers against all nine AHSV serotypes were already detectable on day 15 after the prime immunization. After the boost immunization, nAb titers increased for eight out of nine serotypes, with the highest titers observed for serotypes 3 and 9 (Figure 1D). Significant increases, up to approximately 1:320, were detected for five out of nine serotypes: AHSV-3, -4, -5, -8, and -9 (Figure 1D). For serotypes 2, 6, and 7, mice also showed increased nAb titers after immunization (three out of the five mice), although these increases were not statistically significant. Importantly, the nAb titers achieved against these three serotypes still reached substantial levels, ranging from approximately 1:160 to 1:280, which are considered indicative of a good immune response.

### Characterization of AHSV challenge conditions in IFNAR (-/-) mice

**Figure 2:**
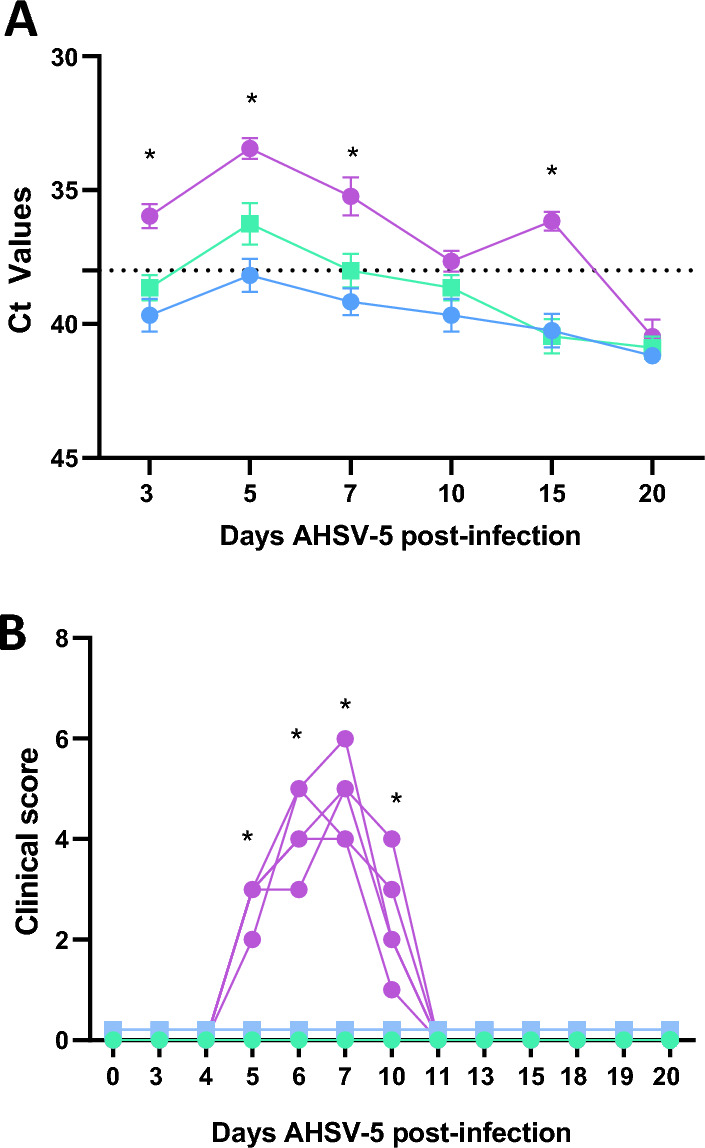
**Inoculation of different doses of rAHSV-5.** Groups of IFNAR(-/-) mice (*n* = 5) were subcutaneously inoculated with 10^5^, 10^4^, 10^3^ PFU of AHSV-5. **A** RNAemia was analyzed by specific RT-qPCR after viral inoculation of IFNAR(-/-) mice at 3, 5, 7, 10, 15 and 20 dpi Results were expressed as Ct (left y axis). Points represent mean Ct values for each mouse group and error lines represent the SEM. “No Ct” values were considered as a Ct of 42. **B** Clinical scores were calculated in mice groups after viral inoculation by using semi-quantitative method described in Table 1. Differences between groups were calculated by multiple t test analysis using the Sidak–Bonferroni method. *P value < 0.05.

The major aim of the work was to test the protective capacity of the DISA cocktail vaccine against different AHSV serotypes. A challenge dose of 10^6^ PFU/mouse of AHSV-4 was used for protection studies in mice, and causes clinical signs and mortality as described in earlier works [36]. It was intended to use a similar dose of AHSV-5 (virus generated by reverse genetics) [42] for lethal infection, but this was not possible as rAHSV-5 did not reach a sufficiently high virus titer. The infective dose of rAHSV-5 in IFNAR (-/-) mice was determined by subcutaneous inoculation of three groups (*n* = 5) with 10^3^, 10^4^ or 10^5^ PFU/mouse. At the highest infectious dose, clinical signs were observed (ruffled hair, reduced activity, weight loss and ocular discharges) but there was no mortality (Figure 2A), while lower doses did not induce signs of illness. Further, viral RNA was detected in blood samples from 10^5^ PFU infected mice from 3 to 15 dpi, with higher values on day 5 post-infection (Figure 2B). However, there were no positive results by RT-qPCR throughout the experiment for the group infected with the lowest dose of rAHSV-5. In contrast, mice receiving the medium dose displayed detectable but significantly lower viral RNA levels compared with those infected with 10^5^ PFU. In summary, significant differences in clinical scores and RNA levels were found between the 10^5^ PFU/mouse and the groups infected with lower doses. We considered that a dose of 10^5^ PFU rAHSV-5 was suitable and was subsequently used in the efficacy experiment to study protection against rAHSV-5 challenge.

### Protection by AHS DISA cocktail vaccine in IFNAR (-/-) mice against AHSV-4 and AHSV-5

**Figure 3:**
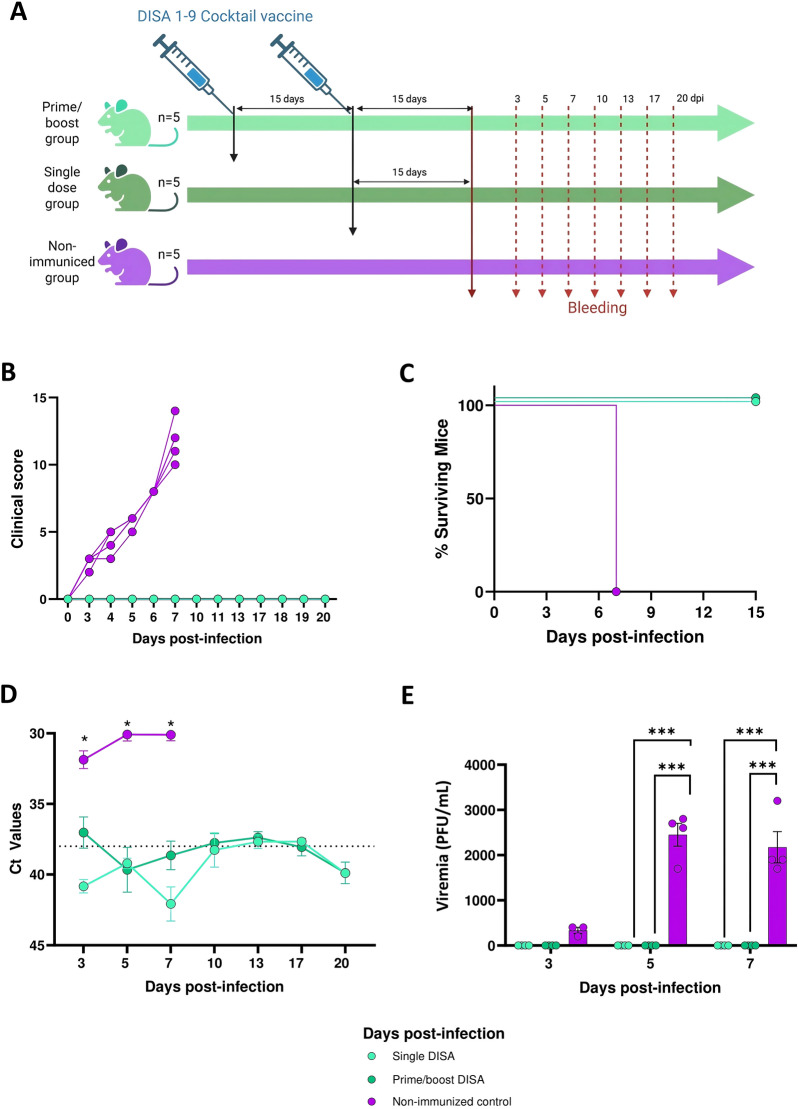
**Evaluation of efficacy of DISA cocktail vaccine against AHSV-4.** **A** Experimental design of the experiment. Groups of mice (*n* = 5) were immunized with DISA vaccines in a single (prime) or prime/boost regime (two weeks apart). Two control groups of mice were mock vaccinated. At two weeks after the prime or the boost, mice were challenged with AHSV-4. Blood samples were collected at 3, 5, 7, 10, 13, 17 and 20 dpi for quantification of RNAemia and viremia. **B** Daily clinical score observed in mice infected with AHSV. Clinical scores were obtained across different clinical signs daily observed after challenge (Table 1) for the time period of 0–20 dpi. **C** Survival rates after challenge. Survival curves were found statistically significant compared with survival curves of control animals as calculated by log-rank test (*P* < 0.05). **D** RNAemia analyzed by RT-CR after challenge. Expression of mRNA was quantified at 3, 5, 7, 10, 13, 17 and 20 days post-infection. Results were expressed as Ct values. Points represent the mean Ct value of each group. Differences between groups were calculated by multiple t test analysis using the Sidak–Bonferroni method. *P < 0.05. and **E** Evaluation of viremia by plaque assays on Vero cells after challenge. Virus titers were calculated as PFU per mL of blood. Two-way ANOVA with a post hoc Tukey test for multiple comparisons was used to calculated statistic differences (*** = *P* < 0.001).

**Figure 4:**
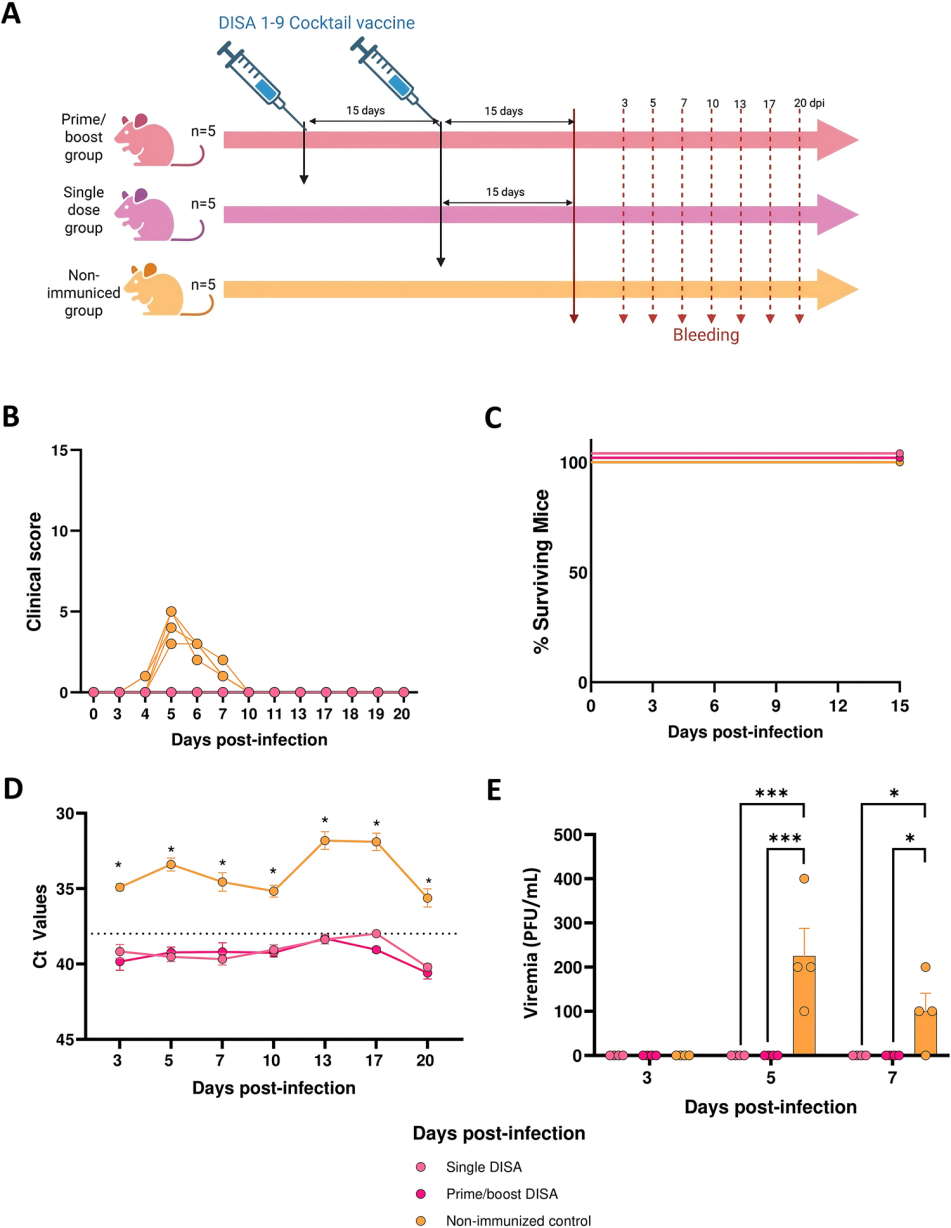
**Evaluation of efficacy of DISA cocktail vaccine against AHSV-5.** **A** Experimental design of the experiment. Groups of mice (n = 5) were immunized with DISA vaccines in a single (prime) or prime/boost regime (two weeks apart). Two control groups of mice were mock vaccinated. At two weeks after the prime or the boost, mice were challenged with rAHSV-5. Blood samples were collected at 3, 5, 7, 10, 13, 17 and 20 dpi for quantification of RNAemia and viremia. **B** Daily clinical score observed in mice infected with AHSV. Clinical scores were obtained across different clinical signs daily observed after challenge (Table 1) for the time period of 0–20 dpi. **C** Survival rates after challenge. Survival curves were found statistically significant compared with survival curves of control animals as calculated by log-rank test (*P* < 0.05). **D** RNAemia analyzed by RT-qPCR after challenge. Expression of mRNA was quantified at 3, 5, 7, 10, 13, 17 and 20 days post-infection. Results were expressed as Ct values. Points represent the mean Ct value of each group. Differences between groups were calculated by multiple t test analysis using the Sidak–Bonferroni method. *P < 0.05. and **E** Evaluation of viremia by plaque assays on Vero cells after challenge with AHSV-4. Levels of infectious virus were calculated as PFU per mL of blood. Two-way ANOVA with a post hoc Tukey test for multiple comparisons was used to calculated statistic differences (*** = *P* < 0.001; * = *P* < 0.05).

To evaluate the protective capacity of the DISA cocktail vaccine, mice were vaccinated with a single dose or prime-boost strategy with 9 × 10^5^ TCID_50_ DISA vaccine in total per vaccination, corresponding to 10^5^ TCID_50_ per DISA vaccine, with a two-week interval between prime and boost vaccination. Two weeks after the booster or after the single dose, mice were subcutaneously challenged with 10^6^ or 10^5^ PFU of AHSV-4 or rAHSV-5, respectively (Figures 3A and 4A).

After challenge with virulent AHSV-4, all control mice developed clinical signs starting at 3 dpi, such as ruffled hair, weight loss, ocular discharges, respiratory alteration and high reduction in mobility. At 7 dpi, all control mice reached the humane endpoint (HEP) and were euthanized (Figure 3B). In contrast, both DISA-vaccinated groups (single or prime/boost vaccinated) did not show clinical signs and all survived to AHSV-4 challenge (Figure 3C). RNAemia was detected in the mock vaccinated animals from 3 to 7 dpi with an average Ct value of 30.1 at 7 dpi (Figure 3D). Infectious virus was detected in blood from these AHSV-4 control mice at 3, 5 and 7 dpi (Figure 3E). In contrast, DISA-vaccinated mice (single or prime/boost) showed average Ct values of > 38 (assay’s cut-off is a Ct value of 38) at most analyzed days post-infection (Figure 3D). Further, infectious virus could not be detected in samples from vaccinated (single or prime/boost) animals after AHSV-4 infection (Figure 3E). The amount of infectious virus on 3 dpi was very low and not statistically different from the negative result for DISA vaccinated animals on 3 dpi, while there were statistical differences at 5 and 7 dpi between vaccinated groups and the control mice.

After AHSV-5 challenge, infected control mice survived, but showed clinical signs such as ruffled hair, ocular discharge and a slight drop in weight (Figure 4B). Both DISA-vaccinated groups also survived and importantly did not present any signs of disease after rAHSV-5 challenge (Figure 4C). Viral RNA was detected from 3 to 20 dpi in the control group. Furthermore, Ct values of all control mice were positive at any analyzed day post-infection with the highest average Ct values at 13 and 17 dpi. In contrast, single and prime/boost vaccinated groups did not show detectable RNA levels above the cut-off Ct value of 38 at any day post-challenge (Figure 3D). In agreement with the RT-qPCR results, infectious virus was not detected in vaccinated groups after challenge, whereas infectious virus was found on 5 and 7 dpi in blood from the control animals (Figure 3E). From 10 dpi onward, no infectious virus was isolated from the blood in any of the groups. Notably, lower viremia and lower amounts of viral RNA were detected in the control mice for rAHSV-5 than for AHSV-4, however the dose of rAHSV-5 was 1log lower compared to AHSV-4.

In conclusion, single or prime/boost vaccination with DISA cocktail vaccine (multivalent AHS DISA-DIVA vaccine for all nine serotypes) completely protected IFNAR (-/-) mice against lethal AHSV-4 and virulent rAHSV-5.

## Discussion

A safe, broad protective AHS DIVA vaccine is the best option to protect horses against this lethal pathogen. LAVs are efficacious, however their safety is questionable and are not DIVA-compatible. Improved safety and broad protection are desperately needed to control AHS in endemic regions. Broad protective, safe AHS DIVA vaccine would be also attractive for countries at high risk of incursion of yet unknown AHSV serotypes. Reverse genetics for AHSV, in line with successes for prototype BTV, has resulted in promising AHS DISA-DIVA vaccine candidates for all nine serotypes, designated DISA1 to DISA9 [46]. These DISA vaccines are based on live attenuated AHSV with a deletion of 77 amino acid codons in Seg-10, which is suitable for accompanying DIVA diagnostics [43, 46]. Safety of DISA vaccines is highly likely, since this region in Seg-10 of highly virulent AHSV-5 is essential for virulence and transmission by midges but not essential for virus replication in vitro [42]. The DISA cocktail vaccine did not cause clinical signs, viremia nor side effects after inoculation of mice. Thus, individual DISA vaccines as well as multivalent DISA cocktail vaccine were demonstrated to be completely safe in IFNAR (-/-) mice. Furthermore, potential reassorted variants of DISA1 to DISA9 will be not virulent, as DISA vaccines share the crucial deletion in Seg-10. Previously, viremia by prototype DISA5, highly virulent AHSV-5 with the deletion in Seg-10, was not detected after prime/boost vaccination of ponies [42]. Here, prime/boost vaccination of mice was also completely harmless and RNAemia was not observed. Taken together, reassortment between DISA vaccine and virulent AHSV in co-infected cells of DISA-vaccinated/infected mice is negligible. DISA1 to DISA9, and more importantly, DISA cocktail vaccine are completely safe in mice, and now justifies experiments in the equine target host to study vaccine safety, immune responses and eventually protection. Here, the pronounced attenuation of DISA vaccines was evidenced by the complete absence of systemic dissemination and detectable blood-borne virus, even in these interferon-deficient mice where viral replication would normally be poorly controlled. These findings suggest that the primary mechanism of attenuation of DISA vaccines is a profound defect or delay in release from primarily infected host cells after vaccination, rather than the absence of NS3/NS3a mediated immunosuppression. Comparative studies with virulent recombinant rAHSV-5 and the derived prototype DISA5 would allow a more direct assessment to unravel NS3/NS3a functions in this mouse model.

Upon efficacy, LAVs closely mimics virus infection by expressing all viral proteins leading to lasting protection [55]. Similarly, DISA-DIVA vaccines in the DISA cocktail vaccine also expose all viral proteins to the immune system, except for the deleted 77 amino acids in NS3/NS3a protein [42]. Notably, DISA-DIVA vaccines lack multifunctional NS3/NS3a protein, which also plays a role in suppression of the host’s immune system by interfering with several pathways [56, 57]. This is an important advantage upon efficacy, since, in addition to serotype specific immunodominant VP2 protein, less immunogenic viral proteins contribute to protection [58–61]. DISA vaccines can undergo multiple rounds of replication, however, are more attenuated than LAV. Indeed, prime/boost vaccination with prototype DISA vaccine for serotype 5, did not cause viremia and did not induce detectable nAbs after prime/boost vaccination [42]. Nevertheless, ponies were partially protected and demonstrated the potential of AHS DISA-DIVA vaccines. Improvement of vaccine efficacy was intended by selecting live attenuated vaccine virus AHSV4LP as backbone to recover AHS DISA-DIVA vaccines for all nine serotypes [46].

Obviously, vaccine studies in the target equine host are needed eventually but face resistance from the community. Particularly, aiming protection for all nine distinct AHSV serotypes is very challenging, requires a huge number of horses and will be extremely expensive. To overcome these hurdles, we successfully studied here the efficacy of multivalent DISA cocktail vaccine in the established IFNAR (-/-) mouse model. Immunization resulted in nAb titers against all nine serotypes (Figure 1D). The overall trend showed boosted responses across eight out of nine serotypes, although increase of nAb titers after boost immunization for AHSV-2, -6, and -7 was not statistically significant at the times analyzed in this study. Generally, these results indicated the platform's broad immunogenic potential. Notably, nAb titers varied between serotypes after both immunizations that could be explained by use of different reference strains for PRNTs leading to technical variation in determined nAb titers for different serotypes. More likely, the antigenicity of outer shell proteins can vary in mice as was also observed in guinea pigs for equal amounts of baculovirus expressed VP2 proteins [26]. Further, DISA vaccines all grow to high virus titers in cell cultures, however, small differences in growth kinetics in vivo and subsequently differences in exposure to the immune system cannot be excluded. DISA vaccines share replication proteins and only differ for the outer shell proteins, which are particularly involved in an early phase (cell entry) of host cell infection. Therefore, variability of boost effects by different DISA vaccines could also be explained by the timing of boost vaccination. Taken together, variability in nAb titers between different serotypes could be expected. Nevertheless, all nine DISA vaccines as present in DISA cocktail vaccine induce nAbs and are all immunogenic in IFNAR (-/-) mice. It is yet unknown what the variability in nAb titers means regarding serotype specific protection.

In general, nAb titers against AHSV are indicative for protection; a nAb titer of 32–64 was determined as the correlation of protection (CoP) for inactivated AHS vaccine [45]. Furthermore correlation between nAb titers and protection has been observed for several vaccine approaches, such as MVA based vaccines [62], canarypox vectors [61] and subunit vaccines [35, 63, 64]. These vaccine candidates mainly focused on protection by exposure of VP2 to the immune response. A nAb titer of 32–64 was determined as the CoP for inactivated AHS vaccine [45]. Nonetheless lack of detectable nAbs does not mean lack of protection per se [42, 65]. Cell-mediated immunity against VP2 as well as immune responses against other viral proteins likely contribute to protection [56, 58, 59, 65–67].

Likely, the CoP vary widely between different types of vaccine. Presumably, the CoP for AHS DISA vaccine as determined for a couple of AHSV serotypes will be indicative for the other AHSV serotypes. Here, prime and prime/boost vaccinated mice were both fully protected against disease and systemic infection from challenge by virulent AHSV serotypes 4 and 5 (Figures 3 and 4). The prime vaccinated group showed similar or lower nAb titers for the serotypes 4 and 5 used in challenge to the other serotypes, AHSV-1 to 3 and 6 to 9 (Figure 1D). Therefore, based on nAb titers for serotype 4 and 5 as CoP suggests protection of IFNAR (-/-) mice against all serotypes after single vaccination with DISA cocktail vaccine. Further, boost vaccination increased nAb titers for most serotypes, which might be important knowledge with regard to future studies in horses.

In this study, rAHSV-5 was not as virulent in the IFNAR (-/-) mouse model than in the natural host [42]. Nevertheless, rAHSV-5 efficiently infected IFNAR (-/-) mice causing clinical signs and viremia and the course of infection was also dose dependent. Thus higher doses will be explored in future infection experiments. Further investigation is needed to characterize differences in the interference mechanisms with innate immune response pathways among AHSV serotypes, as well as to assess potential variation in virus attenuation in mammalian cell cultures used to propagate AHSV strains.

In summary, our study provides compelling preclinical evidence that multivalent DISA-DIVA vaccines represent a safe and promising vaccine candidate to achieve broad protection against AHS. Future work should evaluate efficacy and DIVA of this safe DISA cocktail vaccine in horses, and assess protection, lasting immunity, vector transmission dynamics, and operational deployment in endemic and outbreak scenarios.

## Data Availability

All data generated or analyzed during this study are included in this published article.
